# Apremilast mitigates interleukin (IL)-13-induced inflammatory response and mucin production in human nasal epithelial cells (hNECs)

**DOI:** 10.1080/21655979.2021.1987818

**Published:** 2021-10-18

**Authors:** Jia Liang, RuoXiao Zhuang, XueYao Sun, Feng Zhang, Bin Zou

**Affiliations:** a Department of Otorhinolaryngology, Children’s Hospital of Chongqing Medical University, Chongqing, 400014, China; b National Clinical Research Center for Child Health and Diseases, Ministry of Education Key Laboratory of Child Development and Disorders, China International Science and Technology Cooperation Base of Child Development and Critical Disorders, Children’s Hospital of Chongqing Medical University, Chongqing, 400014, China; c Chongqing Key Laboratory of Pediatrics, Chongqing, 400014, China

**Keywords:** Apremilast, IL-13, allergic rhinitis, mucin, NF-κB

## Abstract

Interleukin (IL)-13-associated inflammatory response is important for the pathogenesis of allergic rhinitis (AR). Apremilast is a phosphodiesterase-4 (PDE4) inhibitor approved for psoriasis treatment. Here, we investigated the potential effects of Apremilast against IL-13-induced injury in human nasal epithelial cells (hNECs). Firstly, Apremilast ameliorated oxidative stress in IL-13-challenged cells by decreasing the levels of reactive oxygen species (ROS) and the production of malondialdehyde (MDA). Secondly, Apremilast inhibited the expressions of IL-6 and IL-8. Moreover, Apremilast inhibited the expressions of the chemokines colony-stimulating factor 2 (CSF2) and chemokine ligand 11 (CCL11). Interestingly, exposure to IL-13 increased the expressions of mucin 4 and mucin 5AC (MUC5AC), which was ameliorated by treatment with Apremilast. Interestingly, we found that Apremilast inhibited the phosphorylation of c-Jun-N-terminal kinase (JNK). Importantly, Apremilast reduced the levels of c-fos and c-Jun, the two AP-1 subfamilies. The luciferase reporter assay demonstrates that Apremilast reduced the transcriptional activity of activator protein 1 (AP-1). Lastly, we found that Apremilast prevented the activation of nuclear factor kappa-B (NF-κB) by decreasing the levels of nuclear NF-κB p65 and the luciferase activity of the NF-κB reporter. In summary, we conclude that Apremilast possesses a protective effect against IL-13-induced inflammatory response and mucin production in hNECs by inhibiting the activity of AP-1 and NF-κB.

## Introduction

Allergic rhinitis (AR) is defined as a chronic nasal mucosal inflammatory disease mediated by immunoglobulin E (IgE). It is characterized by sneezing, itchy, stuffy,, and a watery nose, accompanied by changes in smell and taste [1,2]. A series of complications, such as allergic asthma, sinusitis, nasal polyps, conjunctivitis, and secretory otitis media, are reported in AR, and significantly impact the normal lives of patients [3]. Currently, the involvement of inflammatory cells in the development of AR has been widely reported, including activated B cells [4], Th1 cells, Th2 cells [5], Treg cells [6], and Th17 cells [7]. Cytokines released by these inflammatory cells are regarded as the direct inducer and stimulator of AR, among which IL-13 is an important pro-inflammatory factor released by Th2 cells [8]. It is reported that the production of IL-13 in the peripheral blood of AR patients is significantly elevated, further inducing the local infiltration of eosinophilic granulocytes [9]. In addition, IL-13-stimulated nasal epithelial cells (NECs) are widely used as the evaluation *in vitro* assay for AR in experimental studies [10,11]. Both oxidative stress and inflammation are reported to induce injury to human NECs, contributing to the development of AR [12]. Mucin is the main component of mucus in the nasal mucosa and bronchus, the expression of which is reported to be significantly promoted in chronic inflammatory diseases, such as asthma, cystic fibrosis, and AR [13]. Borchers claimed that the expression levels of mucin 5AC were observed to be greatly upregulated in the respiratory tract of acrolein-stimulated rats [14]. Temann [15] reported that in IL-13 overexpressing mice, non-ciliary cell hypertrophy was induced, accompanied by the dramatically elevated expression level of mucin 5AC. Inflammation is an important mechanism underlying the pathogenesis of AR and is regulated by multiple types of inflammatory pathways. Shi claimed [16] that the activation of the JNK pathway contributes to the excessive production and accumulation of mucin in human NECs. In addition, the role of the AP-1/NF-κB pathway in the development of AR has also been reported [17]. JNK/AP-1/NF-κB pathway is an important regulatory signaling of inflammation, also reported to be involved in the regulation of oxidative stress [18]. Therefore, effective therapeutic methods for the treatment of AR can be achieved by targeting the inflammatory signaling in NECs.

Apremilast is an oral agent for the treatment of psoriasis and psoriatic arthritis developed by Celgene and approved by the US Food and Drug Administration (FDA) in March 2014. It is a specific inhibitor of phosphodiesterase-4 and significantly facilitates the expression of adenosine phosphate by regulating the expression level of phosphodiesterase-4 in immune cells, further contributing to the inhibition of inflammatory reactions [19]. Recently, the anti-inflammatory effects of phosphodiesterase-4 inhibitors, including Apremilast, have been widely reported. However, it is unknown whether Apremilast possesses a beneficial effect in AR. The present study aims to investigate the potential therapeutic effects of Apremilast on AR by investigating the protective property of Apremilast against IL-13-induced damages in NECs.

## Materials and methods

### Cell culture and treatments

Human nasal epithelial cells (hNECs) were purchased from Bnbio (#12,621, Beijing, China) and incubated with the Endothelial Cell Growth Medium-2 (EGM-2) culture kit containing 10% fetal bovine serum (FBS), and cultured in 5% CO_2_ and 37°C. Cells were stimulated with IL-13 (10 ng/mL) [20] in the presence or absence of 2.5 and 5 μM Apremilast (AbMole, Houston, USA) [21] for 24 hours.

### Cell viability measurements

The cell viability of treated human NECs was detected with the cell counting kit-8 (CCK-8) assay. In brief, cells were digested using 0.25% pancreatin and washed 3 times using the PBS buffer, and further seeded in a 96 well plate at 5000/well. Upon the completion of the treatment, cells were loaded with 10% CCK-8 solution. After 2 hours of incubation, the optical density (OD) value at 570 nm was determined (BioTek, Vermont, USA).

### Real-time PCR analysis

The total RNAs were extracted from human NECs treated with a TRIzol reagent (#15,596,026, Invitrogen, California, USA), followed by being transcribed into cDNA using the iScript cDNA synthesis kit (#170-8890 Bio-Rad, California, USA). The PCR reaction conducted in the present study was performed using the SYBR Green Master Mix (#4,385,610, Bio-Rad, USA). Finally, the relative expression level of target genes was determined using the 2^−ΔΔCt^ method after normalization with the expression of GAPDH.

### ELISA assay

The production of IL-6 (#E-EL-H6156 Elabscience, Wuhan, China), IL-8 (#E-EL-H6008 Elabscience, Wuhan, China), CSF2 (#E-EL-H0081c Elabscience, Wuhan, China), CCL11 (#E-EL-H0025c Elabscience, Wuhan, China), and Mucin 4 (#EH1160 Finetest, Wuhan, China) in human NECs was measured with ELISA assay. Briefly, the supernatant of treated NECs was collected and seeded on the 96-well ELISA plate, together with the 5 gradient concentrations of the standard, followed by incubation at 37°C for 1 hour. After removing the medium and 3 washes, the wells were added with the conjugate reagents and the samples were incubated at 37°C for 30 minutes, then TMB solution was added for 15 minutes. Lastly, the stop solution was added and the OD value at 450 nm was determined (Biotek, Vermont, USA).

### Western blot analysis

The total proteins were extracted from cells using the RIPA lysis buffer (50 mM Tris(pH 7.4), 150 mM NaCl, 1% NP-40, 0.5% sodium deoxycholate, 0.1% SDS) (Beyotime Biotechnology, Beijing, China) with the protease and phosphatase inhibitor cocktail [22], which were quantified with a bicinchoninic acid (BCA) kit (#T9300A Takara, Tokyo, Japan). Approximately 30 μg proteins were loaded and separated with 12% sodium dodecyl sulfate-polyacrylamide gel electrophoresis (SDS-PAGE), and further transferred to the polyvinylidene fluoride (PVDF) membrane (Takara, Tokyo, Japan), and then incubated with 5% skim milk. After 3 washes, the membrane was incubated with the primary antibody against p-JNK (1:800, #sc-6254 Santa Cruz Biotechnology, USA), JNK (1:800, #sc-7345 Santa Cruz Biotechnology, USA), c-fos (1:800, #sc-166,940 Santa Cruz Biotechnology, USA), c-Jun (1:800, #sc-74,543 Santa Cruz Biotechnology, USA), NF-κB p65 (1:800, #sc-8008 Santa Cruz Biotechnology, USA), and β-actin (1:800, #sc-8432 Santa Cruz Biotechnology, USA), followed by being incubated with the secondary antibody (1:800, #sc-2005 Santa Cruz Biotechnology, USA) at room temperature for 1.5 hours. Finally, the ECL solution was used to visualize the bands, which were quantified by the Image J software.

### Dihydroethidium (DHE) staining

The level of ROS was determined with DHE staining assay. In brief, cells were incubated with 10 µM DHE at 37°C for 30 minutes, followed by 2 washes with PBS buffer [23]. A laser confocal microscope (Olympus, Tokyo, Japan) was used to visualize the fluorescence intensity and 5 random fields were selected for the calculation of fluorescence expression.

### Malondialdehyde (MDA) measurements

The production of MDA was detected using the commercial MDA Assay Kit (Nanjing Jiancheng Bioengineering Institute, Jiangsu, China) according to the method described previously [24].

### Luciferase activity

Cells were transfected with NF-κB or AP-1 luciferase reporter (GenePharma Biotechnology, Shanghai, China) containing NF-κB or AP-1 response elements with the firefly luciferase gene inserted in the promoter region and the promoter less-null Renilla construct (GenePharma Biotechnology, Shanghai. China) taken as a negative control. Lip3000 (Invitrogen, California, USA) was used as the transfection reagent. The activity of luciferase and Renilla were determined with the Dual-Luciferase Reporter Assay System (Promega, Wisconsin, USA). The measurement of luciferase in each group was normalized with the measurement of Renilla [25].

### Statistical analysis

The data obtained were expressed as mean ± standard deviation (S.D.) and the data analysis was conducted using the GraphPad Prism version 6.0 (GraphPad Software, USA). The Student’s t-test was used to analyze data between 2 groups and the one-way ANOVA method was used for the analysis among groups. P < 0.05 was considered a significant difference.

## Results

Using an *in vitro* model, we investigated the effects of Apremilast on IL-13-induced damages in NECs. Firstly, we tested the cytotoxicity of Apremilast in hNECs. Secondly, we examined the effect of Apremilast on oxidative stress. Thirdly, we investigated whether Apremilast had an inhibitory effect on the expressions of pro-inflammatory cytokines (IL-6 and IL-8) and chemokines (CSF2 and CCL11). Additionally, we assessed the effect of Apremilast on the expression and secretion of Mucin 4 and MUC5AC in IL-13-challenged hNECs. Importantly, we further investigated the involvement of AP-1 and NF-κB signaling in mediating the protective effects of Apremilast against IL-13.

### Cytotoxicity of Apremilast in human NECs (hNECs)

**Figure 1.**
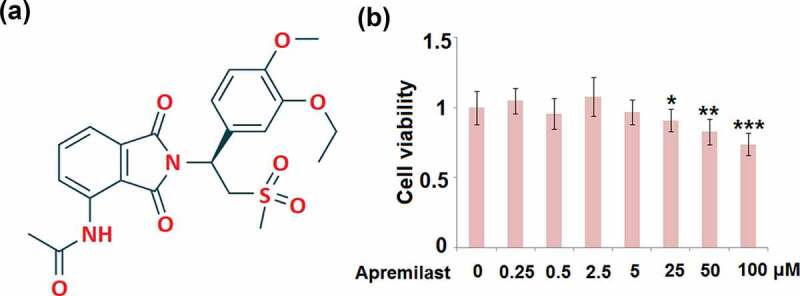
Cytotoxicity of Apremilast in human nasal epithelial cells (hNECs). Cells were stimulated with Apremilast (0, 0.25, 0.5, 2.5, 5, 25, 50, 100 μM) for 24 hours. (a). Molecular structure of Apremilast; (b). Cell viability was measured by the CCK-8 assay (*, **, ***, P < 0.05, 0.01, 0.001 vs. Vehicle group)

To determine the optimized concentrations of Apremilast, NECs were stimulated with different concentrations of Apremilast (0, 0.25, 0.5, 2.5, 5, 25, 50, 100 μM) for 24 hours, followed by detecting the cell viability using the CCK-8 assay. The molecular structure of Apremilast is visualized in Figure 1a. As shown in Figure 1b, as the concentration of Apremilast increased from 0 to 5 μM, the cell viability remained unchanged, which was significantly declined as the concentration of Apremilast exceeded 25 μM. Therefore, in the subsequent experiments, 2.5 and 5 μM were utilized as the optimized concentrations of Apremilast.

### Apremilast attenuated IL-13-induced oxidative stress in hNECs

**Figure 2.**
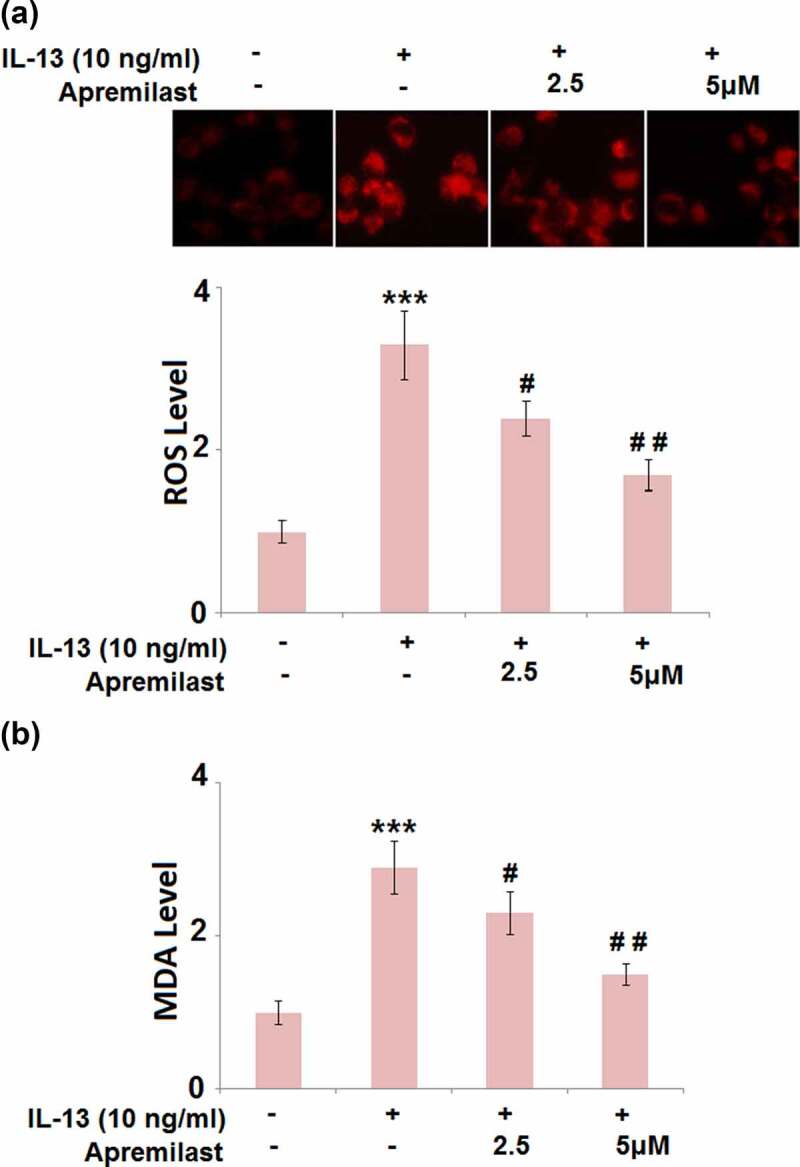
Apremilast attenuated IL-13-induced oxidative stress in hNECs. Cells were stimulated with IL-13 (10 ng/ml) in the presence and absence of Apremilast (2.5, 5 μM) for 24 hours. (a). The levels of ROS; (b). The levels of MDA (***, P < 0.001 vs. vehicle group; #, ##, P < 0.05, 0.01 vs. IL-13 group)

As shown in Figure 2a, compared to the control, the ROS levels were dramatically elevated by stimulation with IL-13 but greatly suppressed by the introduction of Apremilast. In addition, the promoted production of MDA in the IL-13 group was significantly declined by treatment with the 2.5 and 5 μM Apremilast (Figure 2b). These data reveal that the state of oxidative stress in IL-13-treated hNECs was greatly alleviated by Apremilast.

### Apremilast inhibited IL-13-induced expression of pro-inflammatory cytokines in hNECs

**Figure 3.**
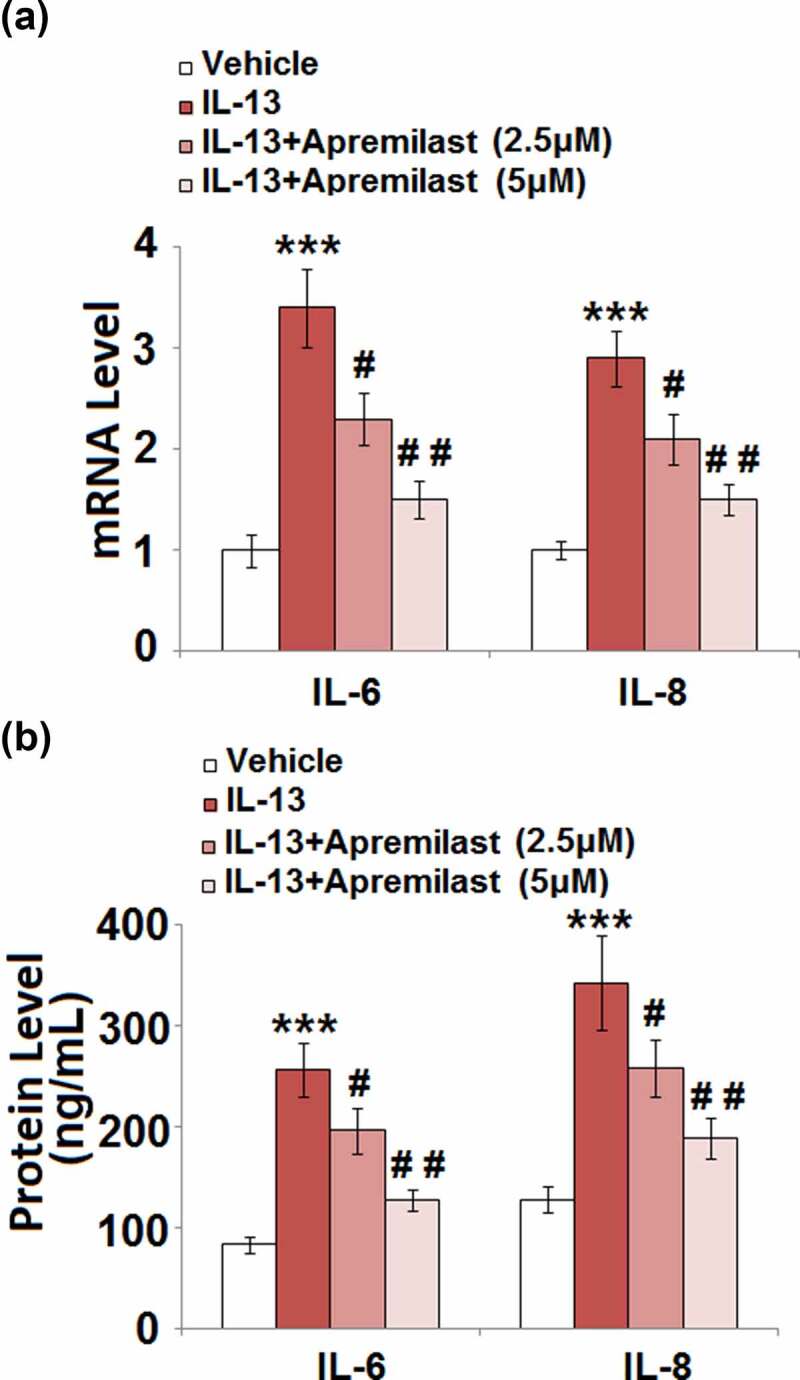
Apremilast inhibited IL-13-induced expression of pro-inflammatory cytokines in hNECs. Cells were stimulated with IL-13 (10 ng/ml) in the presence and absence of Apremilast (2.5, 5 μM) for 24 hours. (a). mRNA of IL-6 and IL-8; (b). Secretions of IL-6 and IL-8 (***, P < 0.001 vs. vehicle group; #, ##, P < 0.05, 0.01 vs. IL-13 group)

Activated inflammation and excessively released inflammatory factors in NECs are important characteristics of AR. As shown in Figure 3a, compared to the control, the gene expression levels of IL-6 and IL-8 were significantly promoted by the stimulation of IL-13, then greatly downregulated by treatment with Apremilast. The production of IL-6 (Figure 3b) in the control, IL-13, 2.5 μM Apremilast, and 5 μM Apremilast group was 83.6, 256.7, 196.5, and 127.4 pg/mL, respectively. In addition, compared to control, the concentration of IL-8 was significantly elevated from 128.1 pg/mL to 342.5 pg/mL IL-13, which was greatly suppressed to 258.7 pg/mL and 189.6 pg/mL by treatment with 2.5 and 5 μM Apremilast, respectively. These data indicate that the activated inflammatory state in IL-13-treated NECs was dramatically mitigated by Apremilast.

### Apremilast suppressed IL-13-induced expression of colony-stimulating factor 2 (CSF2) and CCL11 in hNECs

**Figure 4.**
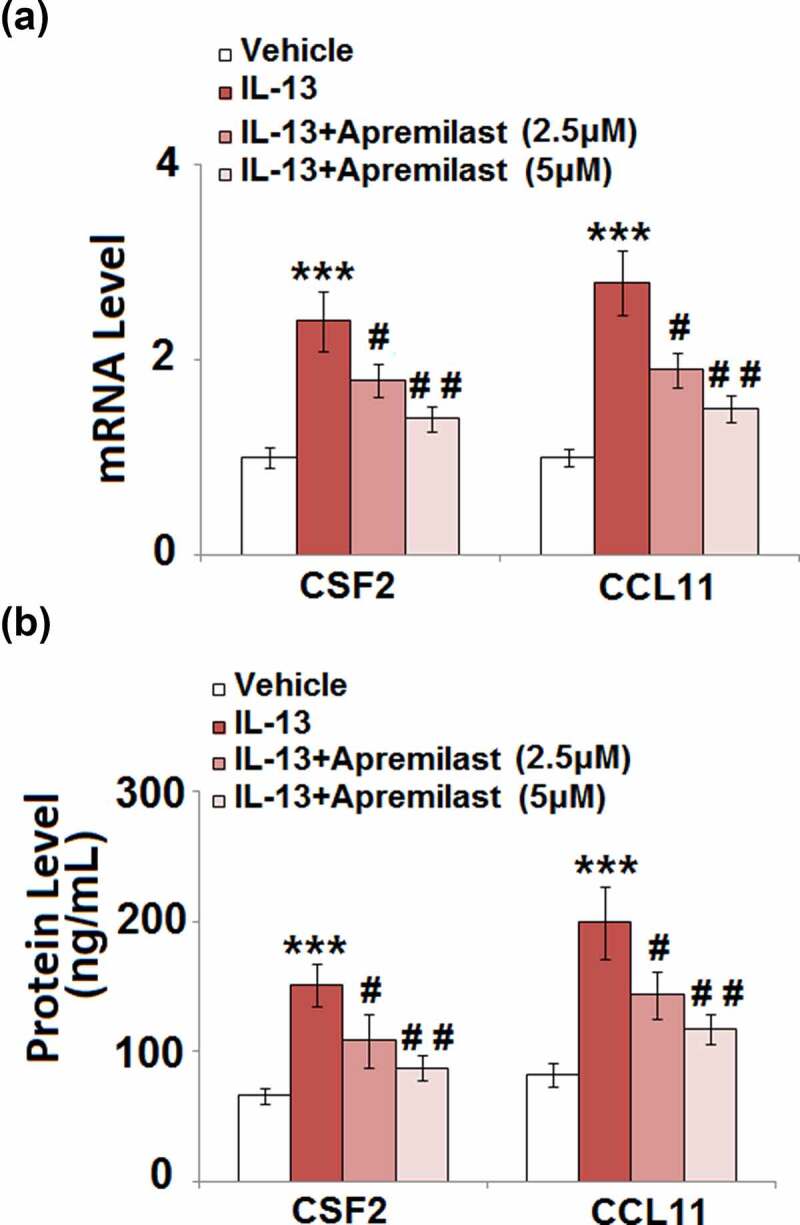
Apremilast suppressed IL-13-induced expressions of CSF2 and CCL11 in hNECs. Cells were stimulated with IL-13 (10 ng/ml) in the presence and absence of Apremilast (2.5, 5 μM) for 24 hours. (a). mRNA of CSF2 and CCL11; (b). Secretions of CSF2 and CCL11 as measured with ELISA (***, P < 0.001 vs. vehicle group; #, ##, P < 0.05, 0.01 vs. IL-13 group)

CSFs and chemokines are important mediators for the recruitment of inflammatory cells, such as macrophages which induce severe inflammation [26,27]. As shown in Figure 4a, the upregulated CSF2 and CCL11 in IL-13 stimulated cells were dramatically reversed by treatment with Apremilast. In addition, compared to the control, the secretion of CSF2 (Figure 4b) was significantly increased from 66.2 pg/mL to 151.4 pg/mL after stimulation with IL-13, then dramatically declined to 108.5 pg/mL and 87.8 pg/mL by the introduction of 2.5 and 5 μM Apremilast, respectively. The concentrations of CCL11 in the control, IL-13, 2.5 μM Apremilast, and 5 μM Apremilast groups were 82.3, 199.5, 143.7, and 117.6 pg/mL, respectively. These data indicate that Apremilast significantly decreased the possibility of inflammatory cells recruitment by downregulating the CSFs and chemokines.

### Apremilast inhibited the expression of Mucin 4 and Mucin 5AC (MUC5AC) in hNECs

**Figure 5.**
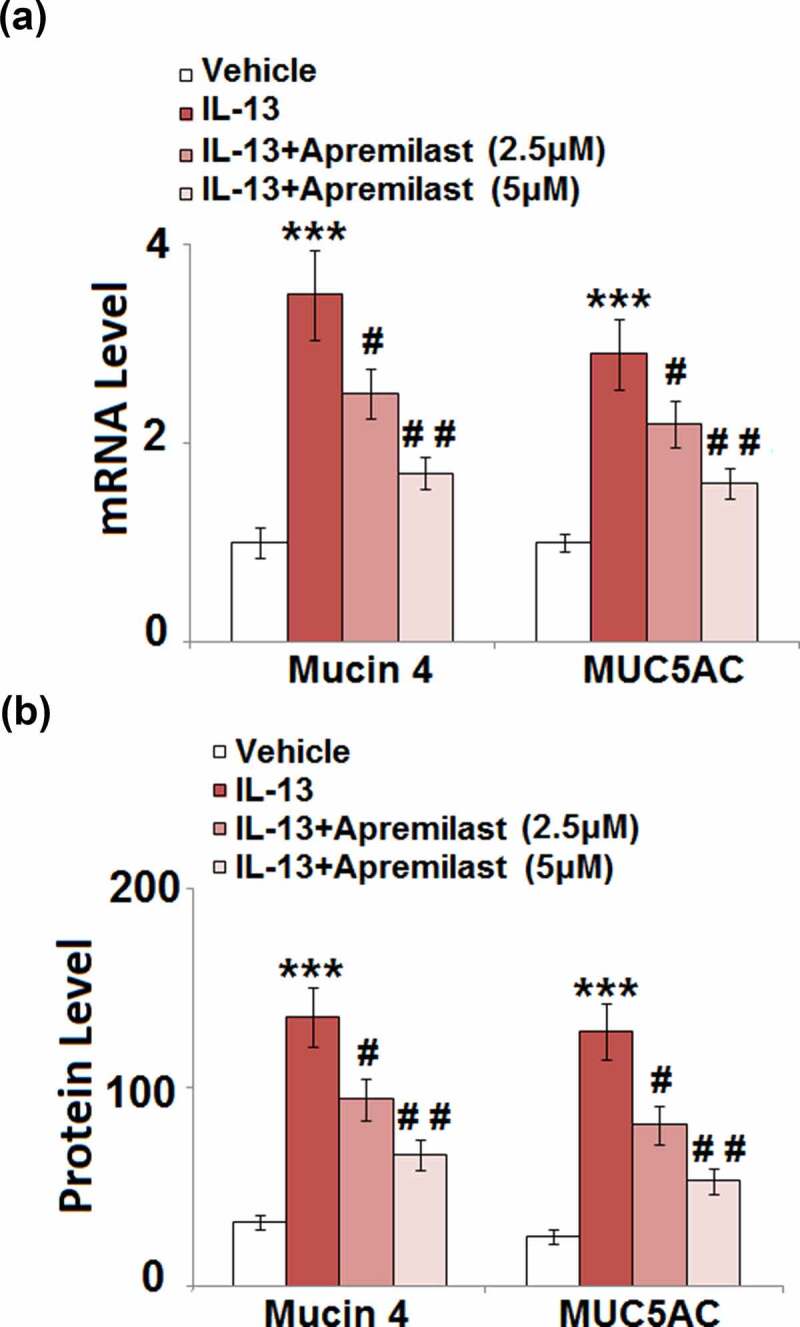
Apremilast inhibited the expression of Mucin 4 and Mucin 5AC (MUC5AC) in hNECs. Cells were stimulated with IL-13 (10 ng/ml) in the presence and absence of Apremilast (2.5, 5 μM) for 24 hours. (a). mRNA of Mucin 4 and MUC5AC; (b). Secretions of Mucin 4 and MUC5AC (***, P < 0.001 vs. vehicle group; #, ##, P < 0.05, 0.01 vs. IL-13 group)

Accumulation of mucin in the nasal mucosa epithelium is the main symptom of AR [28]. The expressions and secretions of mucin 4 and MUC 5AC were further determined after cells were treated with different strategies. As shown in Figure 5a, compared to the control, the expression levels of mucin 4 and MUC 5AC were significantly elevated by treatment with IL-13 but greatly suppressed by the introduction of Apremilast. The secretions of mucin 4 in the control, IL-13, 2.5 μM Apremilast, and 5 μM Apremilast groups were 32.6, 135.7, 94.2, and 66.5 pg/mL, respectively. In addition, compared to the control, the release of MUC 5AC was dramatically increased from 25.1 pg/mL to 128.3 pg/mL by stimulation with IL-13, which was greatly declined to 81.5 pg/mL and 53.2 pg/mL by treatment with 2.5 and 5 μM Apremilast, respectively (Figure 5b). These data indicate that the excessive production of mucin proteins in IL-13-treated NECs was dramatically ameliorated by Apremilast.

### Apremilast mitigated the activation of JNK in IL-13 challenged hNECs

**Figure 6.**
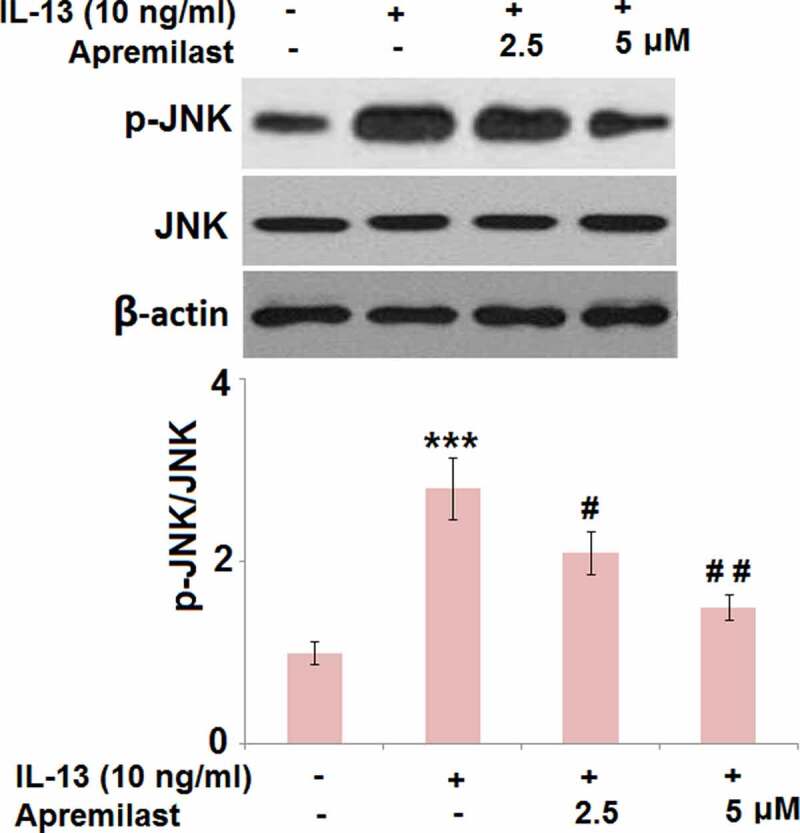
Apremilast mitigated the activation of JNK in IL-13 challenged hNECs. Cells were stimulated with IL-13 (10 ng/ml) in the presence and absence of Apremilast (2.5, 5 μM) for 24 hours. The levels of p-JNK and total JNK were measured by western blot analysis (***, P < 0.001 vs. vehicle group; #, ##, P < 0.05, 0.01 vs. IL-13 group)

JNK is an important inflammatory pathway that can be activated by IL-13 [29]. As shown in Figure 6, compared to the control, the expression level of p-JNK/JNK was dramatically elevated in the IL-13 group but significantly suppressed in the 2.5 and 5 μM Apremilast groups, respectively, indicating an inhibitory effect of Apremilast on the activated JNK pathway in IL-13-treated NECs.

### Apremilast prevented activation of the transcriptional factor AP-1 and NF-κB

**Figure 7.**
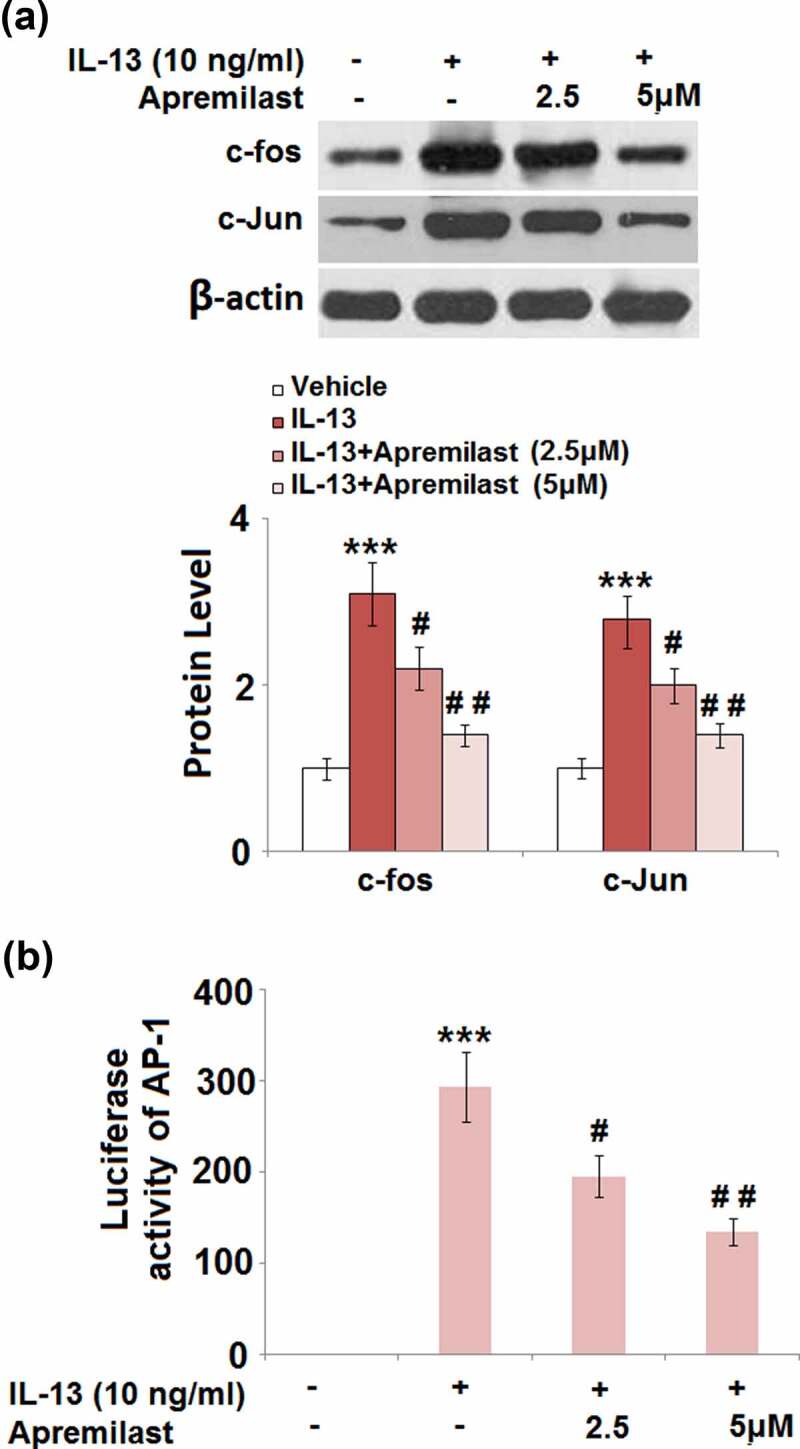
Apremilast prevented activation of the transcriptional factor AP-1. Cells were stimulated with IL-13 (10 ng/ml) in the presence and absence of Apremilast (2.5, 5 μM) for 24 hours. (a). The expressions of c-fos and c-Jun; (b). Luciferase activity of AP-1 (***, P < 0.001 vs. vehicle group; #, ##, P < 0.05, 0.01 vs. IL-13 group)

**Figure 8.**
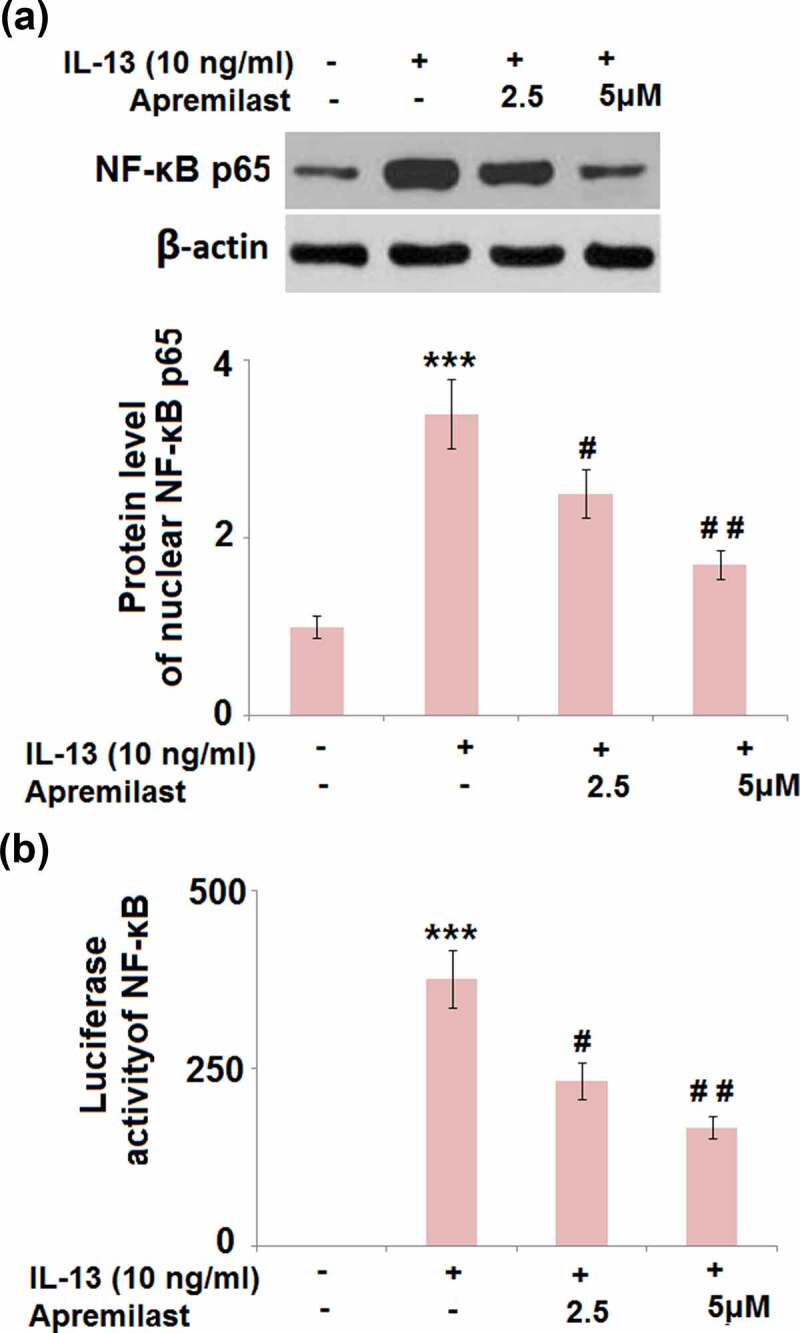
Apremilast ameliorated IL-13- induced activation of the transcriptional factor NF-κB. Cells were stimulated with IL-13 (10 ng/ml) in the presence and absence of Apremilast (2.5, 5 μM) for 24 hours. (a). Protein level of nuclear NF-κB p65;(b)Luciferase activity of NF-κB (***, P < 0.001 vs. vehicle group; #, ##, P < 0.05, 0.01 vs. IL-13 group)

JNK is the upstream activator of AP-1.AP-1 and NF-κB are both important transcriptional factors involved in the regulation of inflammation [30]. As shown in Figure 7a, the elevated expression levels of c-fos and c-Jun in the IL-13 group were greatly suppressed by treatment with Apremilast. In addition, the promoted luciferase activity of AP-1 (Figure 7b) in the IL-13 group was dramatically declined in the 2.5 and 5 μM Apremilast groups. As shown in Figure 8a, compared to the control, nuclear NF-κB p65 was pronouncedly upregulated by stimulation with IL-13, then dramatically downregulated by the introduction of Apremilast in a dose-dependent manner. In addition, the elevated luciferase activity of NF-κB (Figure 8b) in the IL-13 group was significantly decreased in the 2.5 and 5 μM Apremilast groups. These data reveal that the activated inflammatory transcriptional factors in IL-13 treated NECs were dramatically suppressed by Apremilast.

## Discussion

Reactive oxygen species (ROS) play an important role in regulating mucous secretion by activating the transcriptional factors sensitive to redox [31,32]. They are regarded as the intermediate signal transduction molecule in the downstream of IL-13 [33]. Excessive production and accumulation of ROS contribute to redox imbalance, resulting in the activation of oxidative stress [34], accompanied by the elevation of MDA production and declined SOD activity [35]. In the present study, we found that the excessive release of ROS and promoted concentration of MDA were observed in IL-13-treated NECs, indicating the activation of oxidative stress in NECs was induced by IL-13. After treatment with Apremilast, the state of oxidative stress was found to be significantly alleviated.

IL-13 not only stimulates the synthesis of mucin but also induces its secretion [36], which is consistent with IL-13-stimulated upregulation of mucin 4 and MUC 5AC observed in the present study. After the introduction of Apremilast, the accumulation of mucin was dramatically mitigated. The receptor with a high affinity to IgE can be induced and upregulated by IL-13 and the allergen complex can be directly delivered to the specific T cells by these receptors, further enhancing the anaphylaxis. As the main effector for inflammation and tissue restructure located in Th2 cells, IL-13 is reported to coexist with chemokines and their receptors [37]. In the present study, not only was severe inflammation in NECs directly induced by IL-13, the production of chemokines and CSFs was also facilitated, which might recruit inflammatory cells to enhance the inflammation. After treatment with Apremilast, the state of inflammation was significantly alleviated, accompanied by the downregulation of CSFs and chemokines. In our future work, the interaction between NECs and monocytes in the presence of IL-13 and Apremilast will be further investigated to better understand the anti-inflammatory effect of Apremilast.

The promoter of MUC 5AC is reported to be the binding site of both AP-1 and NF-κB [38,39]. JNK can be activated by multiple elements, including LPS, pro-inflammatory factors, and stress. The phosphorylation of c-Jun can be induced by the activated JNK protein, enhancing the transcriptional activity of c-Jun. The phosphorylation of c-Jun on its amino terminal facilitates the formation of the heterodimer of c-Jun/c-Fos and the homodimer of c-Jun/c-Jun, which then binds to the AP-1 site located in the promoter domain of multiple genes to enhance their transcriptional activity [40–42]. The phosphorylation of I-κB, a natural inhibitor of NF-κB, can also be induced by activated JNK, contributing to the transfer of NF-κB into the nucleus. As a consequence, the expression level of NF-κB p65 in the nucleus will be promoted, it will then bind to the promoters of inflammatory factors and facilitate their expressions. In the present study, we found that the JNK/AP-1 and the JNK/NF-κB pathways in NECs were both activated by IL-13, which was dramatically reversed by treatment with Apremilast.

## Limitations

Although this study has attempted to highlight the role of Apremilast in the treatment of AR, there are certain limitations that require future investigation. There was no control group using other drugs licensed for the treatment of AR such as loratadine, to compare the effects of Apremilast in AR. Moreover, *in vivo* experiments are required to further elaborate the protective benefits of Apremilast in AR. The underlying mechanisms involved in the development of AR need further clarification. In our future work, we will design more experiments using both *in vitro* and *in vivo* models to further clarify the underlying mechanisms involved in AR and the protective effects of Apremilast against it. Additionally, a JNK agonist or JNK-overexpressed NECs will be introduced to further verify the regulatory effect of Apremilast on JNK pathways.

## Conclusion

Taken together, our data indicate that Apremilast mitigated IL-13-induced inflammatory response and mucin production in human nasal epithelial cells, suggesting its potential use in the treatment of AR.

## Data Availability

Data and materials of this study are available upon reasonable request to the corresponding authors.
